# Concurrent Measurement of Mitochondrial DNA Copy Number and ATP Concentration in Single Bovine Oocytes

**DOI:** 10.3390/mps4040088

**Published:** 2021-12-07

**Authors:** Casey C. Read, Sadikshya Bhandari, Sarah E. Moorey

**Affiliations:** Department of Animal Science, University of Tennessee, Knoxville, TN 37996, USA; cread6@utk.edu (C.C.R.); sadikshya.bhandari@gmail.com (S.B.)

**Keywords:** single cell analysis, oocyte, ATP, mitochondria, mitochondrial DNA

## Abstract

To sustain energy-demanding developmental processes, oocytes must accumulate adequate stores of metabolic substrates and mitochondrial numbers prior to the initiation of maturation. In the past, researchers have utilized pooled samples to study oocyte metabolism, and studies that related multiple metabolic outcomes in single oocytes, such as ATP concentration and mitochondrial DNA copy number, were not possible. Such scenarios decreased sensitivity to intraoocyte metabolic relationships and made it difficult to obtain adequate sample numbers during studies with limited oocyte availability. Therefore, we developed and validated procedures to measure both mitochondrial DNA (mtDNA) copy number and ATP quantity in single oocytes. Validation of our procedures revealed that we could successfully divide oocyte lysates into quarters and measure consistent results from each of the aliquots for both ATP and mtDNA copy number. Coefficient of variation between the values retrieved for mtDNA copy number and ATP quantity quadruplicates were 4.72 ± 0.98 and 1.61 ± 1.19, respectively. We then utilized our methodology to concurrently measure mtDNA copy number and ATP quantity in germinal vesicle (GV) and metaphase two (MII) stage oocytes. Our methods revealed a significant increase in ATP levels (GV = 628.02 ± 199.53 pg, MII = 1326.24 ± 199.86 pg, *p* < 0.001) and mtDNA copy number (GV = 490,799.4 ± 544,745.9 copies, MII = 1,087,126.9 ± 902,202.8 copies, *p* = 0.035) in MII compared to GV stage oocytes. This finding is consistent with published literature and provides further validation of the accuracy of our methods. The ability to produce consistent readings and expected results from aliquots of the lysate from a single oocyte reveals the sensitivity and feasibility of using this method.

## 1. Introduction

The accumulation of adequate stores of metabolic substrates as well as an increase in mitochondrial number within the oocyte are important components of the acquisition of developmental competence. Increases in mitochondrial number and the creation of metabolic substrate stockpiles occur throughout folliculogenesis [1,2,3,4,5,6,7,8,9,10]. The oocyte and early embryo have low levels of glycolytic activity and rely on mitochondrial oxidative phosphorylation to produce the ATP necessary to sustain development to the blastocyst stage of embryo development [11,12,13,14]. Substrates and co-factors such as pyruvate, acetyl CoA, NADH, CO_2_, and FADH are produced from glycolytic activity of the cumulus cells and transferred to the oocyte via gap junctions to support oxidative phosphorylation [12,15,16]. This transfer of metabolic substrates occurs throughout oogenesis until the onset of oocyte maturation leads to gap junction breakdown. The oocyte must support energy demanding developmental processes like fertilization and embryonic cell division with the mitochondria and metabolic substrates accumulated prior to oocyte maturation.

Previous studies have linked intraoocyte ATP levels, mtDNA copy number, and mitochondrial function to oocyte developmental competence [17,18,19,20,21]. Within bovine oocytes, reported ATP concentrations range from 0.25 to 35 pmol and reported mtDNA levels range from 13,000 to 3,600,000 copies per oocyte. Due to low quantities of starting materials in single oocytes, many studies utilized pooled oocytes for analyses, and no papers have measured multiple metabolic parameters within a single oocyte. Single cell analysis is a valuable tool to better understand and evaluate intraoocyte relationships among metabolic components and mitochondria functionality. While pooling oocytes allows researchers to account for high variability in abattoir-derived oocytes, single oocyte analyses are necessary for in vivo studies where sample numbers are often limiting.

Therefore, we developed a protocol with the objective to divide the lysate of a single oocyte into quarters and measure ATP and mtDNA copy number in duplicate, quarter oocyte equivalents. After successful validation of the protocol for both ATP and mtDNA measurements, we performed a second study with the objective to compare ATP quantity and mtDNA copy number between germinal vesicle (GV) and metaphase two (MII) stage oocytes as an additional validation to demonstrate that the protocol would yield similar results to previously published studies of pooled oocytes.

## 2. Materials and Methods

### 2.1. Collection of Cumulus-Oocyte-Complexes

No live animals were handled for this study. Abattoir-sourced, mixed breed, bovine (Bos taurus) ovaries were obtained for the manual aspiration of follicles and collection of cumulus-oocyte-complexes (COCs). Briefly, COCs were manually aspirated from 3 to 8 mm follicles using a 12 mL syringe and 18-gauge needle. The aspirate was then transferred to a petri dish and searched for COCs. Once located, COCs were placed into oocyte collection media (OCM; M199 with Hanks’ salts, 2% (*v*/*v*) FBS, 2 mmol/L L-glutamine, 50 U/mL penicillin, and 50 μg/mL streptomycin) until searching was completed. Only those COCs with a homogenous cytoplasm and at least 5 layers of cumulus cells were selected for further use. For the validation of procedures, selected COCs were stripped of their cumulus cells by vortexing for 3–5 min in 1 × trypsin, washed through PBS, and immediately snap frozen in 2 μL of PBS for storage at −80 °C until used for assays. For the comparison of GV and MII stage oocytes, COCs were randomly divided and half (*n* = 15) were collected at the GV stage as described above. The remaining COCs were washed in oocyte maturation media (OMM; M199 with Earle’s salts, 10% (*v*/*v*) FBS, 2 mmol/L L-glutamine, 0.2 mmol/L sodium pyruvate, and 50 mg/mL gentamycin) before undergoing maturation for 22–24 h at 38.5 °C in 5% CO_2_ and humidified air. The matured COCs, which represent MII oocytes (*n* = 15), were then collected, denuded, and stored in the same manner as the GV stage oocytes.

### 2.2. TaqMan Primer and Probe Design

Primers were designed using NCBI BLAST according to the bovine mitochondrial genome (Accession Number: NC_006853.1; Table 1). The selected primer pair created a 183 base pair product from base pairs 11,569 to 11,751 of the bovine mitochondrial genome. The probe sequence was located between the forward and reverse primers and ranged from base pairs 11,621 to 11,645. A gradient polymerase chain reaction (PCR) ranging from 50 to 60 °C was performed to determine optimal annealing temperature ranges of the primers.

### 2.3. Validation

#### 2.3.1. Oocyte Lysis and Division

Germinal vesicle stage oocytes were used to optimize and develop the protocol. Following optimization, we validated the protocol’s ability to provide consistent values for ATP and mtDNA copy number assays in quarter oocyte equivalents. Tubes containing single oocytes were centrifuged at 12,000× *g* for 30 s at 4 °C. For ATP quantification (*n* = 5), 8 μL 5 mM Tris-HCl was added to each sample and all samples were heated at 95 °C for 10 min. Thirty microliters of nuclease free water were added to each sample to achieve a final volume of 40 μL. This volume was then divided into four, 10 μL aliquots for the ATP assay. For mtDNA copy number quantification (*n* = 6), 8 μL Tris-HCl were added to each sample and samples were heated at 95 °C for 10 min. Twenty-six microliters of proteinase K (Zymo Research; Irvine, CA, USA) were added to each sample to reach a final concentration of 200 μg/mL. Samples were heated at 55 °C for 30 min followed by heating at 95 °C for 10 min for proteinase K deactivation. The lysate was then divided into four, 9 μL aliquots for use in qPCR analysis of mitochondrial DNA copy number.

#### 2.3.2. ATP Quantification Validation

Samples were processed using the ATP Determination Kit (Life Technologies; Carlsbad, CA, USA) according to the manufacturer’s directions. Each lysate was thoroughly mixed by pipetting, and 10 μL was added to individual wells of a white, 96 well plate (Costar^®^, 96 well, flat bottomed, white polystyrene assay plate, Corning Incorporated, Corning, NY, USA) and combined with 90 μL of assay mix. Luminescence was recorded using the Synergy H1 Microplate Reader (Biotek; Winooski, VT, USA).

#### 2.3.3. mtDNA Copy Number Quantification Validation

Samples were processed using our custom TaqMan primers and probe (Table 1). Each lysate was mixed thoroughly by pipetting and 9 μL was combined with 1 μL of the custom TaqMan primer/probe mix and 10 μL Fast Advanced Master Mix (ThermoFisher Scientific, Waltham, MA, USA) in a MicroAmp^®^ Fast 96-well Reaction Plate (0.1 mL, ThermoFisher Scientific, Waltham, MA, USA; Table S1A). The PCR settings were as follows: 2 min at 94 °C followed by 40 cycles of 10 s at 94 °C, 15 s at 57 °C, and 12 s at 72 °C (QuantStudio3, ThermoFisher Scientific, Waltham, MA, USA; Table S1B).

### 2.4. Germinal Vesicle and Metaphase II Comparison

#### 2.4.1. Oocyte Lysis and Division

**Figure 1:**
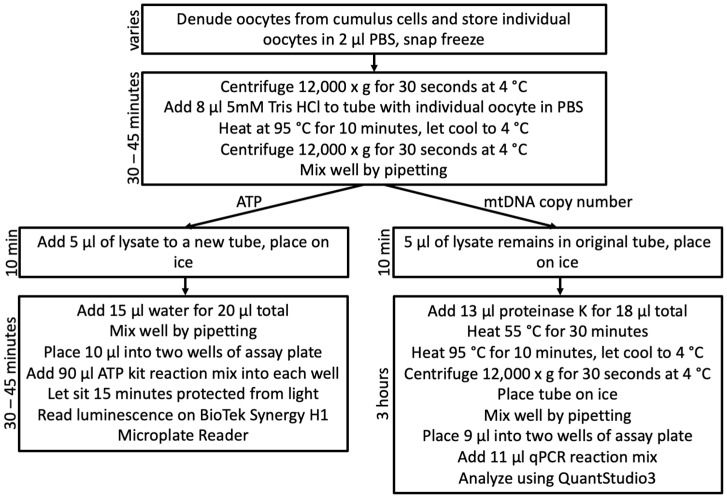
Workflow for oocyte lysis and quantification of ATP levels and mtDNA copy number within a single oocyte.

Tubes containing single oocytes were centrifuged at 12,000× *g* for 30 s at 4 °C. Then, 8 μL 5 mM Tris-HCl was added to each sample and all samples were heated at 95 °C for 10 min. Oocyte lysate was mixed thoroughly by pipetting and 5 μL of oocyte lysate was removed for ATP analysis (Figure 1). Thirteen microliters of proteinase K were added to the tubes with the remaining 5 μL of oocyte lysate to create a final concentration of 200 μg/mL proteinase K. Tubes containing oocyte lysate + proteinase K were heated at 55 °C for 30 min. Proteinase K was deactivated by re-heating at 95 °C for 10 min. After deactivation, the lysate was used for qPCR analysis of mitochondrial DNA copy number (Figure 1).

#### 2.4.2. ATP Quantification

The initial 5 μL of oocyte lysate (collected before proteinase K addition) was diluted with nuclease free water to a total volume of 20 μL which was then divided into two, 10 μL aliquots to perform ATP quantification in duplicate (Figure 1). Standards were generated via dilution of the kit-included 5 mM ATP substrate (0.5 μM, 0.05 μM, 0.025 μM, 0.005 μM, 0.0025 μM). Samples and standards were processed using the ATP Determination Kit (Life Technologies; Carlsbad, CA, USA) as described above. Luminescence values of standards were used to generate a standard curve. The standard curve was used to quantify ATP concentration in each one quarter oocyte equivalent based on the average value of the duplicates assayed for each sample. Oocyte ATP values were then converted to weight using the molarity calculator by GraphPad with the following settings: concentration = micromolar, ATP formula weight = 507.18, volume = 10 μL [22,23]. The result was then multiplied by four because the 10 μL samples used for the ATP concentration assay represented one fourth of an oocyte.

#### 2.4.3. mtDNA Standard Preparation

A group of five oocytes with a homogenous cytoplasm and 3–5 layers of cumulus cells were denuded and snap frozen in 2 μL of PBS. They were then lysed as previously described. The oocyte lysate was then combined with 12.5 μL of Accustart II PCR SuperMix (2×; Quantabio, Beverly, MA, USA), 0.5 μL of 5 μm forward primer, and 0.5 μL of 5 μm reverse primer (Table 1) for a final volume of 25 μL (Table S1C). Cycling conditions of 2 min at 94 °C followed by 40 cycles of 10 s at 94 °C, 15 s at 57 °C, and 12 s at 72 °C were used (Table S1B). The PCR product was further processed through electrophoresis on a 1.5% agarose gel. The resulting band was located at ~183 base pairs, indicating that it was the correct PCR product. The band was then excised from the gel, and the PCR product was purified from the gel using the Zymoclean™ Gel DNA Recovery Kit (Zymo Research, Irvine, CA, USA). DNA concentration of the final eluate was determined using the Qubit™ dsDNA High Sensitivity Assay Kit (ThermoFisher Scientific, Waltham, MA, USA), and purity was determined using 260/280 ratios measured on a NanoDrop spectrophotometer. Number of mitochondrial DNA copies per microliter was calculated from the DNA concentration of the eluate and the molecular weight of the PCR product. The eluate was first diluted 1:1000 before being further diluted to create a standard curve ranging from 10 copies to 1,000,000 copies of the mitochondrial DNA segment per 9 μL. The amplification efficiency of the standard curve was calculated using the qPCR efficiency calculator from ThermoFisher [24].

#### 2.4.4. mtDNA Copy Number Quantification

The 18 μL portion of oocyte lysate and inactivated proteinase K were divided into two, 9 μL aliquots and quantitative PCR (qPCR) was performed in duplicate using our custom TaqMan primers and probe (Table 1). The standards of known mtDNA copy number (described above) were included during each assay. Nine microliters of sample or standard was combined with 1 μL of the custom TaqMan primer/probe mix and 10 μL Fast Advanced Master Mix (ThermoFisher Scientific, Waltham, MA, USA) in a MicroAmp^®^ Fast 96-well Reaction Plate (0.1 mL, ThermoFisher Scientific, Waltham, MA, USA, Table S1A). The PCR settings were as follows: 2 min at 94 °C followed by 40 cycles of 10 s at 94 °C, 15 s at 57 °C, and 12 s at 72 °C (QuantStudio3, ThermoFisher Scientific, Waltham, MA, USA, Table S1B). Average cycle threshold (CT) values were calculated and compared to those obtained from the standard curve to determine mtDNA copy numbers.

### 2.5. Statistics and Analyses

All statistical analyses were performed in R software version 3.6.3 [25]. The corresponding code is available online (https://github.com/CaseyRead/Read_etal_2021_MethdPrtc; accessed on 31 October 2021). For validation of our procedures, coefficient of variation (CV) was calculated for the quadruplicate ATP (fluorescence) or mtDNA copy number (CT) values obtained for each individual oocyte analyzed. The data for ATP and mtDNA values was tested for normality by performing a Shapiro–Wilk test and by plotting the residuals for visual evaluation of normality. The values for ATP were determined to have an approximately normal distribution, while the mtDNA values were natural log transformed. Analysis of variance was performed to determine the differences in ATP and ln(mtDNA) between GV and MII oocytes. Linear regression was performed to determine the relationship between ATP and ln(mtDNA). Results were considered significant if *p* < 0.05. All values are presented as mean ± SD.

## 3. Results and Discussion

### 3.1. Standards Quality

The 260/280 ratio for the PCR product used to make the standards was 1.78. A 260/280 ratio of approximately 1.8 is generally accepted as “pure” for DNA, so we confidently used this PCR product to generate the standards for mtDNA copy number [26,27]. The amplification efficiency of the qPCR assay was calculated to be 100.25%. The desired range for amplification efficiency is 90–110% [28,29,30]. The R^2^ values for the standard curves for each assay were ≥0.99. Desired R^2^ values fall in the range of 0.95–1.0 with the goal to be as close to one as possible [31]. Based on the values for our standard curves falling within the optimal ranges, we are confident that our standard curves were accurate measures of the mtDNA copy number and ATP concentration within the oocyte.

### 3.2. Validation of Procedures for Quantification of ATP and mtDNA Copy Number in Quarter Oocyte Equivalents

We validated that the lysate from a single oocyte could be divided into quarters with minimal variation among the ATP or mtDNA CT values for each quarter oocyte equivalent (Table S2A,B). Intra-assay CV values for ATP and mtDNA were 4.72 ± 0.98% and 1.61 ± 1.19%, respectively. Because the CV values from our methodology were below 5%, we have concluded that there is minimal variation between oocyte lysate portions. This allows us to be confident that the measures obtained via use of this protocol in future studies are valid for comparisons of both ATP and mtDNA copy number in single oocytes.

### 3.3. ATP and mtDNA Copy Number in GV versus MII Oocytes

Of the 30 total oocytes utilized for this study, data from 4 oocytes were removed from the dataset prior to analysis due to values outside of the standard curve. Interassay coefficient of variation was 6.61 ± 5.89 and 2.04 ± 1.48 for the ATP and mtDNA copy number assays, respectively.

**Figure 2:**
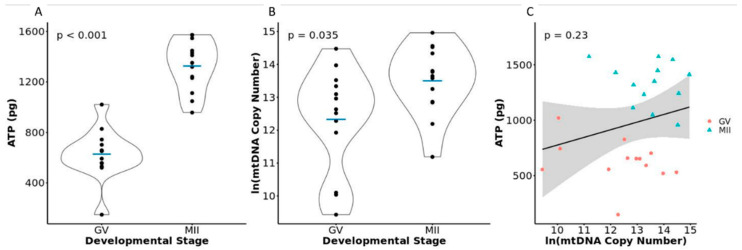
(**A**) Violin plot depicting the differences in ATP quantity within single GV stage and MII stage oocytes. (**B**) Violin plot depicting the natural log of the differences in mtDNA copy number between single GV stage and MII stage oocytes. (**C**) Scatterplot depicting the relationship between the ATP quantity and natural log of the mtDNA copy number within individual oocytes.

Metaphase II stage oocytes had significantly higher quantities of ATP than GV stage oocytes (GV = 628.02 ± 199.53 pg, MII = 1326.24 ± 199.86 pg, *p* < 0.001, Figure 2A, Table S2C). The increased levels of ATP in MII oocytes are consistent with previously published studies of bovine oocytes [1,2,3]. This further validates the accuracy and sensitivity of our protocol to distinguish ATP quantities between different developmental stages of oocytes. However, our values for the concentration of ATP within the oocyte were higher than those previously reported in the literature [1,2,3,20,32,33,34,35]. This difference could be due to differences in assay sensitivity, oocyte dilution factors, and/or additional sources of variation in sample processing. Due to differences in assay methods and dilutions, and to allow for interstudy comparison, we suggest presenting the amount of ATP present within the oocyte as a weight measure.

Mitochondrial DNA copy number was significantly different between GV and MII stage oocytes (GV = 490,799.4 ± 544,745.9, 1,087,126.9 ± 902,202.8, *p* = 0.035, Figure 2B, Table S2C). Multiple studies have shown that, in the bovine, MII stage oocytes have significantly more copies of mtDNA than GV stage oocytes [3,32]. Additionally, the values retrieved for our assessment of mtDNA copy number were in the same range as those previously reported for bovine oocytes [3,4,20,32,34]. This experiment also validated the capability of our methods to measure a large range in mtDNA copy numbers (12,478 to 3,141,658 copies).

Mitochondrial DNA copy number and ATP content were not significantly correlated in our samples (*p* = 0.18, Figure 2C, Table S2C). Iwata et al. 2011 evaluated ATP levels and mtDNA copy number from separate pools of oocytes collected from cows of increasing age and reported a positive correlation between ATP concentration and age, but a negative correlation between mtDNA copy number and age [3]. Such results suggest a negative correlation between ATP concentration and mtDNA copy number. This was the only paper we identified that compared mtDNA copy number to ATP level within the bovine oocyte. One potential reason for our different outcomes is that Iwata et al. 2011 utilized different pools of oocytes for analysis and our measures were from the same oocyte which allowed us to more accurately relate ATP levels to the corresponding mtDNA copy number from the same oocyte. Studies involving other cell types have shown no relationship between ATP content and mtDNA copy number as well as both positive and negative correlations between the two values [21,36,37]. The highly variable relationship between ATP level and mtDNA copy number suggests that there are multiple variables within the cell that are affecting ATP levels and highlights the importance of collecting multiple metabolic measurements when possible to fully elucidate the cause of altered oocyte metabolism.

## 4. Conclusions

Due to small sample volumes, many oocyte-focused studies rely on pooled oocyte samples to have adequate substrate to perform analyses. As technology has advanced, single cell oocyte analysis is possible [38]. Because ATP concentration and mitochondrial numbers within oocytes are important for downstream developmental competence, it is integral that protocols are developed to further investigate these parameters within single oocytes. This would eliminate the need to pool oocytes and retrieve average values for an oocyte population. By retrieving values for individual oocytes, one can relate ATP and mtDNA copy number values, use fewer samples for analysis, and more accurately depict intraoocyte variations in these values.

## Figures and Tables

**Table 1:** Relevant information for primers and probe designed to the bovine mitochondrial genome.

Name	Sequence	Product Length	Tm
mtDNA Primer Forward	CCTACAAACGCTCCTTCCAC	183	59
mtDNA Primer Reverse	AGAGAATATAGGGCGGTGATTACT	183	59
TaqMan Probe	TTGTTGGGGGTAGAGCTAAGTTGGT	---	64

## Data Availability

The data presented in this study are available in Table S2.

## Supplementary Materials

The following are available online at https://www.mdpi.com/article/10.3390/mps4040088/s1, Table S1: Pertinent information for PCR assays, Table S2: Data for validation and comparison of germinal vesicle and metaphase two oocytes.
